# Opportunities drive the global distribution of protected areas

**DOI:** 10.7717/peerj.2989

**Published:** 2017-02-15

**Authors:** Germán Baldi, Marcos Texeira, Osvaldo A. Martin, H. Ricardo Grau, Esteban G. Jobbágy

**Affiliations:** 1 Instituto de Matemática Aplicada San Luis, Universidad Nacional de San Luis & CONICET, San Luis, Argentina; 2 Departamento de Métodos Cuantitativos y Sistemas de Información, Facultad de Agronomía, Universidad de Buenos Aires, Buenos Aires, Argentina; 3 Instituto de Investigaciones Fisiológicas y Ecológicas Vinculadas a la Agricultura, Universidad de Buenos Aires & CONICET, Buenos Aires, Argentina; 4 Instituto de Ecología Regional, Universidad Nacional de Tucumán & CONICET, Horco Molle, Argentina

**Keywords:** Protected areas, National parks, Conservation paradigms, Representativeness, Opportunity, Preferentiality

## Abstract

**Background:**

Protected areas, regarded today as a cornerstone of nature conservation, result from an array of multiple motivations and opportunities. We explored at global and regional levels the current distribution of protected areas along biophysical, human, and biological gradients, and assessed to what extent protection has pursued (i) a balanced representation of biophysical environments, (ii) a set of preferred conditions (biological, spiritual, economic, or geopolitical), or (iii) existing opportunities for conservation regardless of any representation or preference criteria.

**Methods:**

We used histograms to describe the distribution of terrestrial protected areas along biophysical, human, and biological independent gradients and linear and non-linear regression and correlation analyses to describe the sign, shape, and strength of the relationships. We used a random forest analysis to rank the importance of different variables related to conservation preferences and opportunity drivers, and an evenness metric to quantify representativeness.

**Results:**

We find that protection at a global level is primarily driven by the opportunities provided by isolation and a low population density (variable importance = 34.6 and 19.9, respectively). Preferences play a secondary role, with a bias towards tourism attractiveness and proximity to international borders (variable importance = 12.7 and 3.4, respectively). Opportunities shape protection strongly in “North America & Australia–NZ” and “Latin America & Caribbean,” while the importance of the representativeness of biophysical environments is higher in “Sub-Saharan Africa” (1.3 times the average of other regions).

**Discussion:**

Environmental representativeness and biodiversity protection are top priorities in land conservation agendas. However, our results suggest that they have been minor players driving current protection at both global and regional levels. Attempts to increase their relevance will necessarily have to recognize the predominant opportunistic nature that the establishment of protected areas has had until present times.

## Introduction

**Figure 1:**
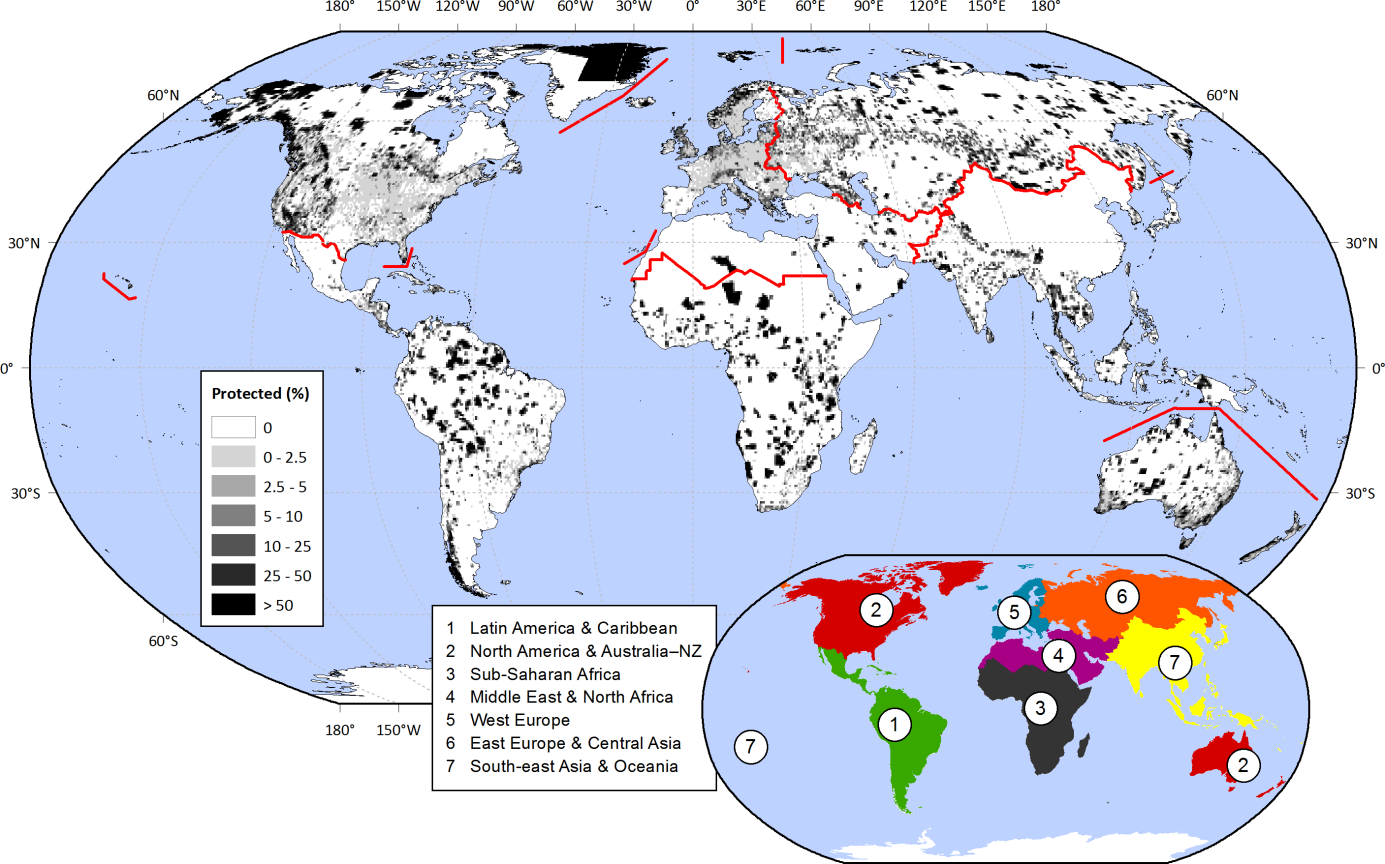
Protected areas fraction on a 0.5° × 0.5° cell basis. IUCN & UNEP-WCMC (2013) data was summarized within 0.5° × 0.5° contiguous cells, considering IUCN categories I–IV (1994). The regions under analysis are depicted in the inset map, and with red lines in the main map. Regional protected fractions are shown in Table S2.

Historically and throughout the world, societies have set aside land from its conventional uses in order to protect particular natural or cultural values (McNeely, Harrison & Dingwall, 1994). In this way, hilltops, old-growth forests, or seashores maintained their biodiversity, scenic attributes, or provision of ecological services. In the last century, simultaneously with the rising pressures over land resources (Vitousek et al., 1997; Ellis et al., 2013), protected areas have greatly increased in number and total area. From just a small handful of locations at the end of the 19th century to thousands nowadays, protection encompasses 15.4% of the world’s continental surface (1.4 × 10^8^ km^2^), excluding Antarctica (Fig. 1) (Juffe-Bignoli et al., 2014).

The current distribution of protected areas responds to a deliberate process guided by a complex interplay of motivations related to perceived societal benefits (McNeely, Harrison & Dingwall, 1994; Pressey, 1994; Margules & Pressey, 2000; Watson et al., 2014). The strength of different motivations changed through history and across territories (Wirth, 1962; Sellars, 1997; Erize, 2003; Mace, 2014). Many of the protected areas established in the late 19th and early 20th centuries responded to practical interests such as favoring tourism or preserving iconic landscape features. However, since the second half of the 20th century, protection has been influenced by a widespread agreement on the importance of maintaining nature in general and biodiversity in particular. Therefore, part of the present-day expansion of protected areas aims to include areas of high species richness, endemism hotspots, or underrepresented ecological or biophysical conditions. Ultimately, we classify these motivations as preferential or representative. The former corresponds to the preservation of specific biological, spiritual, economic, or geopolitical values offered by some territory. The latter corresponds to the protection of a balanced sample of the multiple biophysical environments hosted by a territory, a country, or the whole globe (Pressey, 1994; Lovejoy, 2006) (Table 1). These two groups of motivations interact with different opportunistic forces that shape conservation, as protected areas are frequently deployed in areas that face little human interventions and have comparatively low opportunity-costs, at least at the time of their establishment (Joppa & Pfaff, 2009; Aycrigg et al., 2013; Durán et al., 2013). Consequently, protection has been biased towards unproductive or isolated areas (e.g., cold, dry, with poor soils), leaving other territories inadequately protected despite their potential conservation value (e.g., temperate, subhumid areas) (Pressey, 1994; McNeely & Schutyser, 2003; Hoekstra et al., 2005; Joppa & Pfaff, 2009).

**Figure 2:**
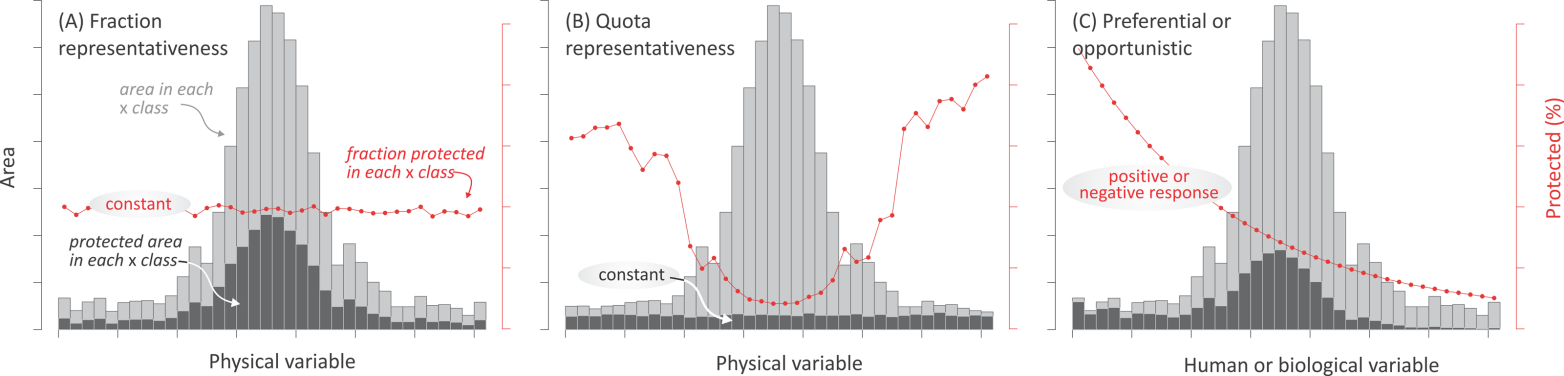
Expected protection patterns according to different forces. Expected geographic patterns of protected areas according to the three groups of forces. In (A) and (B) “fraction” and “quota” representativeness motivations, in (C) preferential motivation and opportunistic forces. Encircled text refers to the expected and tested behavior. Three measurements are shown in the histograms: the area in each class of the independent variables (light gray bars), the area under protection in each *j* class (intervals in the histograms) of the independent variable (dark gray bars), and the fraction under protection of the *j* class of the *i* independent variable (red dots and lines). Only the last two measures were used in the statistical analyses.

Most research about the spatial distribution of protected areas has focused on evaluating the effectiveness of existing networks to encompass biodiversity (Scott, 1993; Brooks et al., 2004; Rodrigues et al., 2004a; Rodrigues et al., 2004b) and biogeographical (ecoregions, biomes, realms) (McNeely, Harrison & Dingwall, 1994; Jenkins & Joppa, 2009; Barr et al., 2011; Watson et al., 2014) or anthropogenical units (Martin et al., 2014). However, few studies have addressed the relative importance that different forces may have had on the deployment of protected areas (Joppa & Pfaff, 2009). Here, we characterized the current distribution of terrestrial protected areas explicitly designated for nature protection—i.e., categorized as I–IV under IUCN guidelines (1994)—in relation to biophysical, human, and biological variables (Table 2). By associating these variables to representative motivations, preferential motivations, and opportunistic forces (Table 1), we assessed the relative impact of these drivers at regional and global levels. While motivations and opportunistic forces are likely to coexist, the predominance of any of them should result in a singular spatial pattern of land protection: (i) If representative motivations prevail, two alternative patterns can be expected, depending on whether protection targets a uniform fraction or on a uniform absolute area of biophysical environments. A uniform fraction leads to a prevalence of the most abundant environments (hereafter, “fraction representativeness”) (Fig. 2A). Alternatively, a uniform absolute area leads to a balanced contribution of common and rare environments (hereafter, “quota representativeness”) (Fig. 2B); (ii) If preferential motivations prevail, protection should be geographically biased towards areas with high biological, spiritual, economic, or geopolitical values (e.g., species diversity, frontiers) (Fig. 2C); (iii) Finally, biases would also arise if opportunistic forces prevail, with protected areas having greater chances of being established where productive potential (e.g., agriculture) and/or human presence are low (Fig. 2C). Our analyses included linear and non-linear regressions, correlations, random forests, and evenness metrics, taking advantage of available spatial datasets.

**Table 1:** Motivations and opportunistic forces related to the implementation of protected areas, sorted by the appearance in history. Acronym: National Park, NP.

Group and name	Origin	Description	Examples
**Preferential motivations**			
Cultural and spiritual	Anthropocentric and non-utilitarian. Early formation of unified societies (e.g., feudal).	Protection is established on remarkable natural and/or cultural sceneries, as their aesthetic appreciation—through direct contact—ensures the fulfillment of basic human needs and thus the well-being of individuals and societies (Bhagwat & Rutte, 2006; Loreau, 2014).	Current Uluru-Kata Tjuta NP (Australia, 1958), Forêt de Fontainebleau (France, 1861)
Gaming and wildlife managing	Anthropocentric and utilitarian. Early.	Protection limits hunting wildlife with the aim of maintaining healthy animal populations (especially ‘singular’ species) and—in the case of gaming—providing recreation to a restricted part of society (Szafer, 1973).	Białowieża Forest (Poland/Belarus, <1541), Pongola Game Reserve (South Africa, 1894)
National imaginary	Anthropocentric and non-utilitarian. Consolidation of modern states.	Similar to the Cultural and spiritual motivation, but with a planned governmental aim of shaping a national pride and identity through natural or cultural icons (Paül Carril, Santos Solla & Pazos Otón, 2015).	Iguazú/Iguaçu NP (Argentina/Brazil, 1935/1939)
Frontier protection and peace preservation	Anthropocentric and utilitarian. Post-independence.	Protection is established close to international borders, as these can be conceived as areas where assert sovereignty or as neutral zones fostering or dedicated to cooperative and peaceful economic activities (Zbicz & Green, 1997).	Waterton-Glacier International Peace Park (Canada/USA, 1932)
Ecosystem goods and services provision	Anthropocentric and utilitarian. Early, but the service concept was popularized since 1900.	Protection is established on territories able to supply over time critical environmental goods and services (timber, water, pollination, soil protection, carbon sequestration) (Costanza et al., 1997).	Malleco National Reserve (Chile, 1907)
Tourism, leisure and recreation	Anthropocentric and utilitarian. Beginning of the 20th century.	Similar to the cultural or spiritual motivation, but with the aim of providing popular entertainment and enjoyment and bringing significant economic benefits to local to regional economies (McKercher, 1996; Mulholland & Eagles, 2002). Could be considered a particular ecosystem service.	Abel Tasman NP (New Zealand, 1942), Nikkō NP (Japan, 1934)
Biological conservation	Biocentric and non-utilitarian. Beginning of the 20th century, but actively after 1960.	Protection is established on territories of high species richness, rates of endemism, or of unique species assemblies (Myers et al., 2000; Terborgh & Winter, 1983).	Virunga NP (Congo DR, 1925), Komodo NP (Indonesia, 1980)
**Representative motivations**			
Fraction	Idem biological conservation	Protection is focused on the representation of ecosystems (biota and processes) due to their intrinsic values (Kareiva & Marvier, 2003; Pressey, 1994), or as pristine scenarios where knowledge of the Earth system can be improved (Bourlière, 1962). Under this motivation, protection targets a uniform fraction of the biophysical environments of a given territory (McNeely, Harrison & Dingwall, 1994; SCBD, 2010), assuming a close relationship between biophysical and ecosystem diversities (Belbin, 1993; Holdridge, 1947).	
Quota	Idem biological conservation	Idem fraction representativeness, but protection targets a uniform absolute area of biophysical environments.	
**Opportunistic forces**			
	Anthropocentric. Beginning of the 20th century.	Protection is established on where opportunity exists, mostly where it is economically feasible, i.e., territories that have a low economic value for traditional and profitable land uses (Margules & Pressey, 2000).	Northeast Greenland NP (Denmark, 1974)

## Methods

### Data sources

The location of protected areas was obtained from the “World Database on Protected Areas”, Annual Release 2013 (IUCN & UNEP-WCMC, 2013). We considered only terrestrial areas explicitly designated for nature protection, i.e., strict nature reserves, wilderness areas, national parks, natural monuments or features, and habitat/species management areas—categories I–IV (IUCN, 1994). We compiled a database of 15 biophysical, human, and biological variables (Table 2). These variables can be directly related to individual motivations and opportunistic forces. For example, the metric “distance to frontiers” can be linked to the preferential motivation of “frontier protection and peace preservation.” We excluded the Antarctica from all analyses.

**Table 2:** Variables related to motivations and opportunistic forces. List of 15 biophysical, human, and biological independent variables, and their relation to the motivations and opportunistic forces of Table 1.

Variable	Calculation and source	Summarizing method	Group and name
Temperature	Mean annual values in °C, from the “Ten Minute Climatology data base” (New et al., 2002), representing averaged monthly figures for the 1961–1990 period.	Mean	Representativeness motivations (fraction and quota)
Precipitation	Amount of annual precipitation in mm. Same source as temperature		
Precipitation to potential evapotranspiration ratio (PPT:PET)	Mean annual values describing water availability (unitless). Same source as temperature. Potential evapotranspiration is retrieved from the Penman-Monteith equation (Allen et al., 2004) and calculated on a monthly basis.		
Elevation	From “Shuttle Radar Topography Mission” (SRTM) digital elevation model (USGS, 2004). Spatial resolution: 90 m. In m above sea level.		
Terrain slope	From “Shuttle Radar Topography Mission” (SRTM) digital elevation model (USGS, 2004). In degrees.		
Soil fertility	Represented by top-soil total exchangeable bases (TEB, 0–30 cm), in cmolc * kg^−1^. From ISRIC-WISE—Global data set of derived soil properties (v.3.0) (Batjes, 2006). Spatial resolution: 30 arc-min.		
Tourism attractiveness	“Panoramio” photos (http://www.panoramio.com) to population counts ratio, in photos * inh^−1^. Modified from the “World touristiness map” (http://www.bluemoon.ee). Panoramio photos were downloaded in December 2013 and processed with Python v.2.7. Population came from the same source referred previously.		Preferential motivations: *Cultural and spiritual*; *National imaginary*; *Tourism, leisure and recreation*
Distance to frontiers	Considering exclusively cells within countries with terrestrial political frontiers. Euclidean distance in km from vector data from “Natural Earth” (http://www.naturalearthdata.com). Cartographic scale: 1:50 m.		Preferential motivations: *Frontier protection and peace preservation*
Biomass	Biomass carbon stored in above and belowground living vegetation circa 2000 (Ruesch & Gibbs, 2008), in Mg ha^−1^. Spatial resolution: 1 km.	Maximum, representing attainable conditions	Preferential motivations: *Ecosystem goods and services provision*
Animal richness	Number of breeding bird, amphibian, and mammal species from Jenkins, Pimm & Joppa (2013). Spatial resolution: 10 km.	Mean	Preferential motivations: *Biological conservation*
Vascular plant richness	Number of vascular plant species from Kreft & Jetz (2007) (combined multipredictor model). Spatial resolution: 110 km.		
Population	Inhabitants from the “Gridded Population of the World v.3 (GPWv3): Population Grids” for the years 1990–1995 (CIESIN-CIAT, 2005). Spatial resolution: 2.5 arc-min.	Sum	Opportunistic forces
Isolation	From the 2000 map “Travel Time to Major Cities” (Nelson, 2008). Representing the distance to large cities (>50,000 inh) in minutes by using a cost-distance algorithm. Spatial resolution: 0.5 arc-min.	Minimum, representing human context of the surrounds of protected areas	
Distance to coasts	Considering ocean coasts. Potentially related to the proximity to docking ports. Euclidean distance in km from vector data from “Natural Earth.”	Mean	
Cropland suitability	Land suitability for low input level rain-fed crops, considering cereals, soybean, and oil palm (FAO/IIASA, 2011). Calculated as the maximum suitability of the included species, per pixel (unitless). Spatial resolution: 5 arc-min.		

### Sampling procedure

We explored the distribution of protected areas at global and regional levels, considering “Latin America & Caribbean,” “North America & Australia–NZ” (New Zealand), “Sub-Saharan Africa,” “Middle East & North Africa,” “West Europe,” “East Europe & Central Asia,” and “South-east Asia & Oceania” (Fig. 1). This regional division relied on cultural, historical, and biogeographical factors (adapted from McNeely, Harrison & Dingwall, 1994; Inglehart & Welzel, 2005; Ellis & Ramankutty, 2008). In order to analyze the links between protection and biophysical, human, and biological conditions (Table 2), we summarized all data into 66,555 cells of 0.5° × 0.5° (Table S1), excluding those with a terrestrial fraction <5%. Compared to other approaches in which each protected area is treated as a single sample, this grid-based approach offered the advantages of (i) providing a unified spatial resolution for all variables, (ii) encompassing the full range of global biophysical, human, and biological conditions, and (iii) avoiding the averaging of these conditions within very large protected areas. Additionally, (iv) this approach provided a clearer representation of the geographical context of protected areas by characterizing the full grid cell in which they are embedded and not just the protected territory (99.55% of the cells incorporates unprotected conditions).

### Data analysis

After summarizing all data within grid cells, we generated 120 histograms —i.e., (7 regions + globe) * 15 independent variables—, containing three sets of information: (i) the absolute area under protection in each *j* class (interval in the histograms) of the *i* independent variable —AREA.PROT—, (ii) the fraction under protection of the class *j* of the *i* independent variable —FRAC.PROT—, and (iii) the area in each class of the *i* independent variables —AREA—; considering a weighted arithmetic mean according to a maximum cell area within each *j* class. For each independent variable, we set a particular class width considering data distribution at the global level. In order to avoid long tails in the histograms, lower and upper *j* classes were grouped using the percentile values 0.025 and 0.975 of the *i* independent variable. At the regional level, we maintained the width of classes in order to facilitate comparisons. We conducted all statistical analyses with the AREA.PROT and FRAC.PROT information separately, while AREA information was shown only for descriptive purposes. For all tests, we carried out the modeling with the values of ≥8 histogram intervals (if not, we divided histogram classes up to accomplish this rule).

We assessed the reciprocal associations between the *i* independent variables through a Kendall’s *τ* non-parametric test (Whittaker, 1987). All calculations were run in RStudio v.0.98.507 (packages Segmented, Scatterplot3d) and Python v.2.7 (packages Scikit-learn, Pandas, Numpy). In order to explore the relative significance of “fraction” and “quota” representativeness motivations (Figs. 2A–2B), we analyzed the existence of a relationship between the FRAC.PROT or AREA.PROT values and the six biophysical variables (Fig. 2) by means of a modification of the “Shannon evenness” (${H}_{i}^{{^{\prime}}}$) (Hill, 1973), calculated as:

(1) \begin{eqnarray*}{V}_{ij}& = \frac{{x}_{ij}}{\sum _{i=1}^{n}{x}_{ij}} \end{eqnarray*}

(2) \begin{eqnarray*}{H}_{i}& =\sum _{i=1}^{n}{V}_{ij}\cdot \ln \nolimits {V}_{ij}\end{eqnarray*}

(3) \begin{eqnarray*}{H}_{i}^{{^{\prime}}}& = \frac{{H}_{i}}{n\cdot \min \left( {V}_{ij}\cdot \ln \nolimits {V}_{ij} \right) } \end{eqnarray*}

where *x*_*ij*_ represents the FRAC.PROT and AREA.PROT in the *j* class of the *i* independent variables, and *n* the number of classes on the histogram. *V*_*ij*_ is calculated to transform FRAC.PROT and AREA.PROT into probabilities. In ${H}_{i}^{{^{\prime}}}$, the numerical effects of an uneven number of classes as well as of despair *x*_*ij*_ magnitudes are canceled. The index ranges between 0 and 1, with a value of 1 when *x*_*ij*_ is constant along the *i* gradient.

While the modified Shannon evenness index indicates the presence of a relationship, regression analyses characterize the behavior of a relationship in terms of shape, sign, and eventually multivariate strength. In this sense, we regressed the FRAC.PROT on the *i* independent variables related to preferential motivations (e.g., animal richness) and opportunistic forces (e.g., cropland suitability) (Fig. 2C). We assessed first and second order polynomials, exponentials, one phase associations, semi-logarithmic (*X* axis logarithmic, *Y* linear), and piecewise models (Faraway, 2006), selected models through the Akaike’s information criterion (Akaike, 1974), and calculated a pseudo-R ^2^ by correlating observed and predicted values from each model as a goodness-of-fit measurement.

We then ranked the relative importance of these variables by means of a random forest algorithm —a machine-learning technique (Breiman, 2001). Random forest estimates the variable importance by looking at how much the mean square error (MSE) increases when the out-of-bag data (observations which are not used for building the current tree, OOB) for that variable are permuted while all others are left unchanged (Liaw & Wiener, 2002). For each unpruned (fully grown) tree, the MSE on the OOB portion of the data is recorded, and then the same is done after permuting each independent variable. Differences between MSE and OOB are averaged over all trees and normalized by their standard deviation. The allocated variable importance can differ substantially with the selection of number of trees to grow (*ntree*), the minimum size of the terminal nodes (*nodesize*), or the number of input variables at each split (*mtry*) (Grömping, 2009; Genuer, Poggi & Tuleau-Malot, 2010). This last parameter has been described as the most critical one; if *mtry* = 1, the splitting variable would be determined completely randomly; whereas a *mtry* =*p* (maximum number of variables) would eliminate the previously described first aspect of randomness, and the possibility of some independent variables—related to the dependent variable but correlated to a stronger regressor—to become the basis of splitting. A usually recommended value on a regression is *mtry* =*p*∕3 because a lower correlation between individual trees improves prediction accuracy (Liaw & Wiener, 2002). However, as the *mtry* values depend on the model and the correlation between independent variables (Breiman, 2001; Grömping, 2009), we set *mtry*^1^ 1For Latin America & Caribbean, North America & Australia–NZ and Sub-Saharan Africa *mtry* = 5; for the globe and East Europe & Central Asia *mtry* = 4; for Middle East & North Africa and West Europe *mtry* = 3; and for South-east Asia & Oceania *mtry* = 2.values that minimize the OOB-MSE of the model (and a *ntree* = 500, and a *nodesize* = 1). The variable importance was used here with an explanatory and interpretative, rather than predictive, aim (Grömping, 2009). We excluded biophysical variables from the random forest since their importance would not reflect the importance of the representativeness motivation, but quite the opposite.

**Figure 3:**
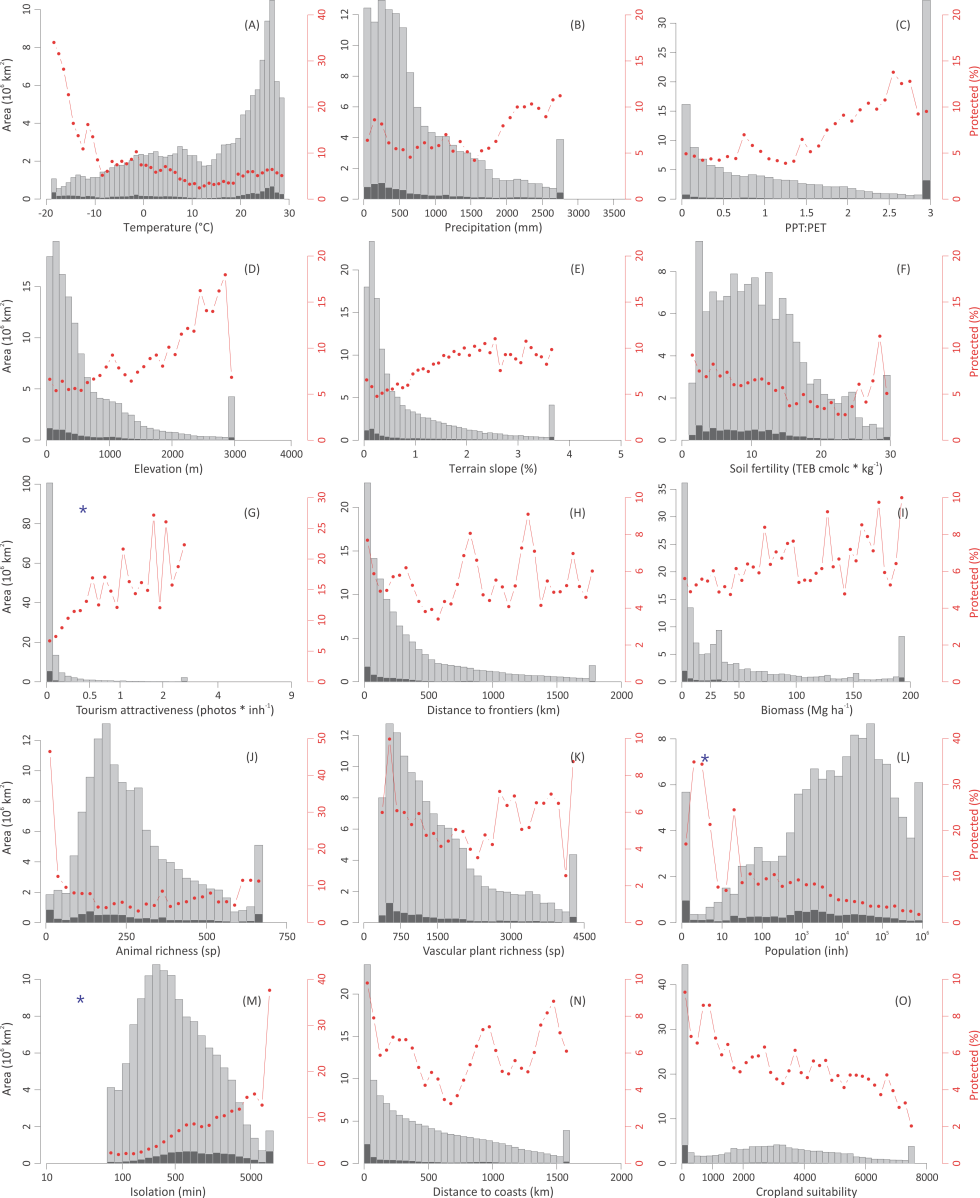
Global distribution of protected areas. Global distribution of protected areas along biophysical, human, and biological gradients. See graphic explanations in Fig. 2. Variables represented in (A–F) are related to the representativeness motivations, those in (G–K) are related to the preferential motivations, and those in (L–O) are related to the opportunistic forces. Lower and upper *j* classes were grouped using the percentile values 0.025 and 0.975 of the *i* independent variable. Blue asterisks denote that histograms were generated with the log_10_ transformed independent variable, and thus do not correspond to the untransformed data used for statistical analyses. Region-specific histograms are shown in Fig. S2.

**Table 3:** Variable importance according to a random forest. Relative importance of nine variables related to opportunistic and preferential motivations (and their grouped averages), according to the random forest. The importances of animals and vascular plants (depicting the single “biological conservation” motivation) were averaged in order to compute the average relative importance of the preferential motivations group.

		Global	Latin America & Caribbean	North America & Australia–NZ	Sub-Saharan Africa	Middle East & North Africa	West Europe	East Europe & Central Asia	South-east Asia & Oceania
**Preferential**	Tourism attractiveness	12.72	0.82	6.93	6.28	9.29	4.87	4.06	3.62
	Distance to frontiers	3.42	10.77	5.94	6.60	4.09	5.52	7.76	19.68
	Biomass	7.31	5.40	12.25	5.58	3.46	4.81	8.40	17.87
	Animal richness	9.19	6.15	0.14	13.52	36.13	10.93	7.18	11.35
	Vascular plant richness	2.54	5.16	0.15	5.03	10.79	10.12	7.28	15.88
	Average	7.33	5.66	6.32	6.93	10.08	6.43	6.86	13.70
**Opportunistic**	Population	19.93	19.72	15.96	11.93	17.17	14.59	6.04	6.73
	Isolation	34.61	47.40	14.18	34.12	5.33	12.65	21.10	8.92
	Distance to coasts	7.22	2.83	4.81	12.37	11.36	3.76	16.99	13.07
	Cropland suitability	3.07	1.76	39.63	4.58	2.38	32.74	21.19	2.89
	Average	16.21	17.93	18.65	15.75	9.06	15.94	16.33	7.90

## Results and Discussion

Globally, opportunistic forces prevailed over preferential and representative motivations in predicting current protection patterns, as protection notably increased towards areas that are isolated, lightly populated, and have low cropland suitability (these three variables are highly correlated, Fig. 3 and Fig. S1). According to the random forest analysis, on average, the importance of the variables related to opportunistic forces doubled in significance those related to preferential motivations (Table 3). These results support previous global explorations that highlighted the importance of opportunistic forces at an ecoregional- (Loucks et al., 2008) or a national- basis (Joppa & Pfaff, 2009). At a regional level, opportunistic forces predominated in North America & Australia–NZ (driven by cropland suitability) and Latin America & Caribbean (driven by isolation) (Table 3 and Figs. S2 and S3). These results challenge Loucks et al’s 2008 realm-based assessment, which showed that globally the number of endemic species was the best variable predicting protected area coverage. Opportunistic forces can lose strength with time (e.g., by road expansion or improvements in crop resistance to biophysical constraints), weakening the legal status of a protected area, a phenomenon of significant magnitude in North America & Australia–NZ, and emergent at the global level (Mascia & Pailler, 2011).

Beyond the imprint of opportunistic forces, protection appeared to respond to preferential motivations that provide benefits to individuals or societies (economic, geopolitical, spiritual). In particular, we found that the tourism attractiveness of an area (Table 2) was positively related to its level of protection (Fig. 3, Figs. S2 and S3), achieving a top importance in the ranking of variables (Table 3). Probably, tourism and protection are involved into positive feedbacks, as protection itself attracts visitors interested in remarkable natural or cultural landscapes, and visitors drive protection to preserve this quality. Tourism engages local communities and regional and national governments in the preservation of these landscapes, offering economic revenues that eventually exceed those obtained from traditional land uses (Mulholland & Eagles, 2002; Siikamäki et al., 2015). As examples, visitors generate annually US$ 1.5 × 10^9^ in the highly populated UK’s Lake District National Park (helping to maintain the landscape naturalness, UK National Parks, 2015). Under a contrasting economic/environmental context, visitors generate annually US$ 2.1 × 10^7^ in the parks inhabited by mountain gorillas in Congo DR, Rwanda, and Uganda (Maekawa et al., 2013). While disentangling the type of existing relationship between tourism and protection is difficult, it is important to note that the most exceptional natural and cultural landscapes around the world are protected under different IUCN categories.

In the last three decades, the inclusion of new species into protection networks as well as the balancing of geographical asymmetries (Stattersfield et al., 1998; Myers et al., 2000; Olson & Dinerstein, 2002) occupied a central place in national and international conservation agendas. However, these motivations are only weakly reflected in the current distribution of protected areas, perhaps because they lead to protection when land is economically unproductive or remote, but not when land is productive and accessible (Margules & Pressey, 2000). Interestingly, new areas created specifically to protect unrepresented environments or species tend to be of small size (Marinaro, Grau & Aráoz, 2012). As a measure on how biological conservation is weakly related to protection, we found that the more intensely protected lands were the poorer in animal and vascular plant species both globally and regionally (Fig. 3 and Fig. S2). Animal and vascular plant richness were positively correlated to cropland suitability at the global level (Kendall’s *τ* = 0.26 for animals and 0.40 for plants, Fig. S1) revealing how the conflict between this biocentric preference and traditional or profitable land uses exacerbates the current biodiversity crisis (Rodrigues et al., 2004a; Rodrigues et al., 2004b; Hoekstra et al., 2005; Venter et al., 2014). The single exception to these findings appeared in South-east Asia & Oceania (Fig. S2), most likely due to the considerable protected systems in highly-diverse countries like Bhutan, Thailand, Cambodia, and Sri Lanka (Fig. 1) (though see Sodhi et al., 2004). Loucks et al. (2008) found a negative relationship between species richness and protection only at the global level and for the Neotropical realm, but not for the remaining five realms, a discrepancy with our results probably related to their ecoregional approach vs. our grid-based approach.

**Figure 4:**
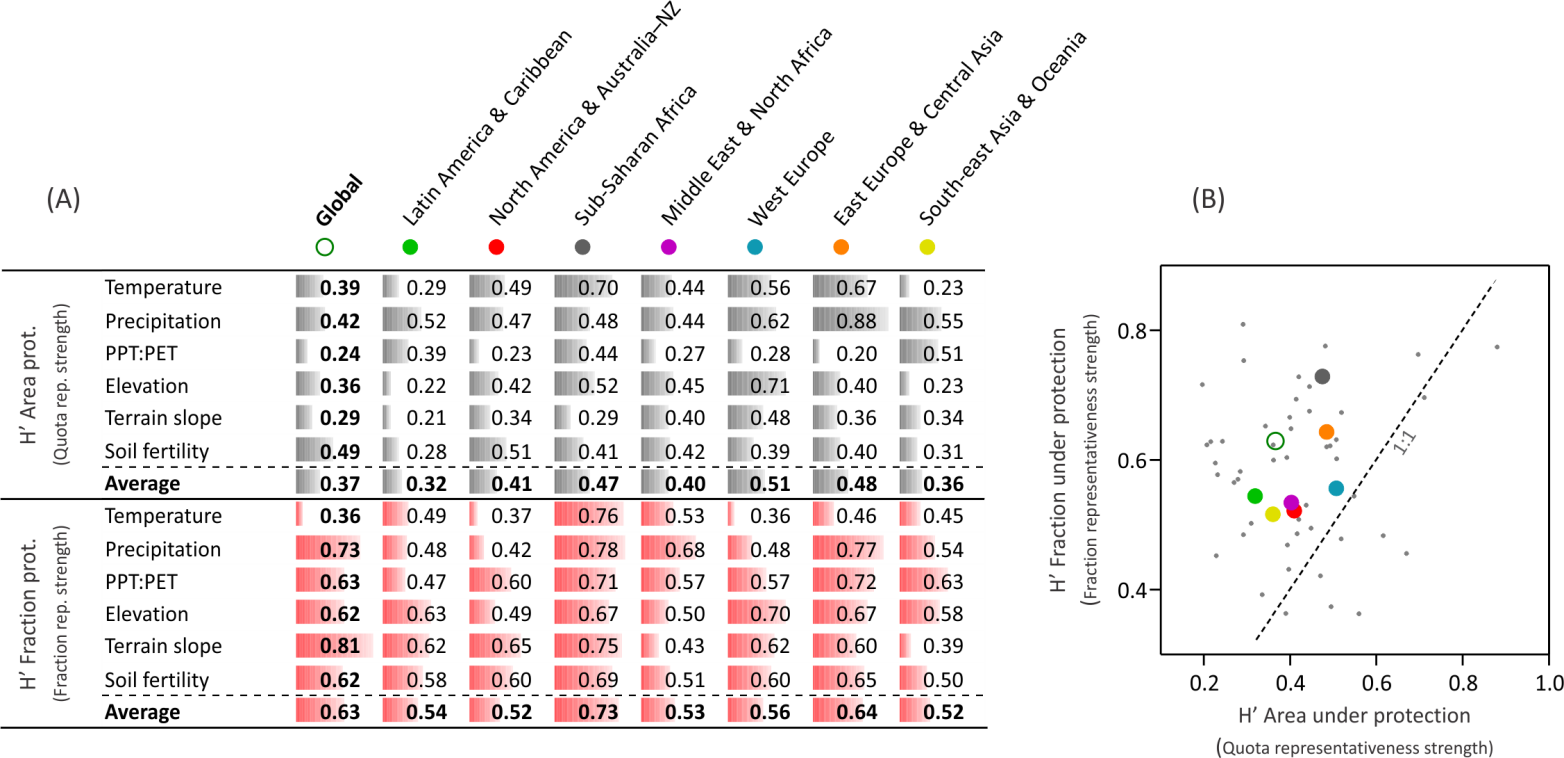
Representativeness according to a modified Shannon evenness. Modified Shannon evenness (*H*′) for the biophysical variables. The index ranges between 0 and 1, with a value of 1 when *x*_*ij*_ is constant along the *i* gradient. (A) *H*′ values of the area under protection (in light gray), related to the “quota representativeness” motivation; and *H*′ values of the fraction under protection (in red), related to the “fraction representativeness”. (B) plot of all H’ values of the 48 biophysical variables * globe/regions combinations (small gray dots), and averaged *H*′ values for the globe and the seven regions (large colored dots).

Representativeness remains nowadays unachieved, as shown by the large biases in the distribution of protected areas along biophysical gradients, with an overrepresentation of lands with extreme climates (polar, arid or very humid), high elevations, complex topographies, and unfertile soils (e.g., Northeast Greenland NP, Denmark; Tassili n’Ajjer NP, Algeria; Fig. 3 and Fig. S2). Even under this context, protection followed closer a fraction- rather than a quota representativeness (1.7-times higher, Fig. 4) according to the modified Shannon evenness index, implying that the current network of protected areas encompasses the most abundant environments. The regions that followed a fraction representativeness more closely were Sub-Saharan Africa and, to a lesser extent, East Europe & Central Asia, while the highest quota representativeness was accomplished by West Europe (Fig. 4). The representativeness levels were lower and similar in the remaining regions, despite the strong differences in their total protected fraction, which ranged from 2.1% in Middle East & North Africa to 11.4% in North America & Australia–NZ (Fig. S2 and Table S2).

Independently of which type of representativeness prevailed, the analysis of protection along biophysical gradients offers the chance to assess the achievement of national and international protection targets and agreements. Among them, the influential Convention on Biological Diversity stipulates that >17% of terrestrial “areas” (i.e., biogeographical units) needed to be included in protected systems by 2020 (SCBD, 2010). Our analyses based on continuous biophysical gradients (which purposely avoid predefined geographical units) show that this protection target is far from being uniformly achieved across the whole array of global environments if we consider exclusively protected areas categorized as I–IV (IUCN, 1994). Regionally, only North America & Australia–NZ in terms of relief, and West Europe in terms of temperature accomplished this protection target (Table S3). The analyses of land protection along biophysical gradients implemented in this study could be used as well to model and to plan future environmental representativeness under a scenario of climate change (Davis & Shaw, 2001; Scott, Malcolm & Lemieux, 2002). The role of unconsidered protected areas (categorized as V “Protected Landscape/Seascape” and VI “Protected area with sustainable use of natural resources” by IUCN—1994) in representativeness remains to be explored.

The predominance of fraction representativeness in the conservation agendas (and in the literature) implies that the environments or geographic units of small extent are unintentionally penalized. The concept of quota representativeness introduced here overcomes this problem, broadening what an equal representation should be. In fact, quotas are often considered in political organization, as many countries have formal electoral rules which warrant a minimum participation of minorities (e.g., ethnic, gender) or an equal contribution of subnational to national administrative entities regardless of their population size (Bird, 2014). This complementary quota conservation approach would ultimately overcome the long-lasting conservation dilemma of hotspot/species-richness vs. coldspot/species-poorness (Myers et al., 2000; Kareiva & Marvier, 2003), as each environment has per se an equal importance (including its encompassed biological distinctiveness and evolutionary strategies).

Regional differences in the weight of alternative motivations and opportunistic forces likely reflect the interactions among direct drivers (e.g., conservation agendas), that—following Lambin, Geist & Lepers (2003)—can be conceptualized as:

Motivations and opportunistic forces =*f* (policies and economy, social organization, moral rules); with

• policies and economy =*f*(agendas, economic/financial contexts, property rights, state-owned lands, infrastructure, governance);
• social organization =*f*(urban-rural interactions, ONG and philanthropists actions);
• moral rules =*f*(importance of religion, priority to environmental protection, deference to authority, trust and tolerance, economic/physical security);

with the functions *f* having variable forms at the time of the establishment of protection. Even though direct drivers have been previously linked to the protected fraction on a country-basis (McDonald & Boucher, 2011), very few studies formulated or assessed their interactions with motivations (Marinaro, Grau & Aráoz, 2012), identifying an adequate fraction representation with strong economies, “modern” societies or states, or extensive and long lasting protection networks. However, North America & Australia–NZ and West Europe, representing these conditions with a pioneering and profuse history of protection (Table S2), were surpassed in the fraction representativeness by other regions, and—at the same time—surpassed others in terms of the weight of opportunistic forces (especially cropland suitability) and preferential motivations (especially tourism attractiveness). The strength of tourism attractiveness in these two regions (Table 3 and Figs. S2, and S3) may reflect the combination of an affluent population capable of devoting resources to “luxury” goods and services (in this case, conservation; Marinaro, Grau & Aráoz, 2012) and a growing need to access natural settings by highly urbanized societies (Pyle, 2003).

With an opposite socioeconomic context, Sub-Saharan Africa reached the top of the representativeness ranking, perhaps due to the historical indirect effect of colonial regimes, unconstrained by the local social organization and with conservation agendas decoupled from local population needs and wills (Naughton-Treves, Holland & Brandon, 2005). In this regard, Sub-Saharan Africa showed the highest fraction of protected areas established before the formation of modern states (Table S2). Historical factors can be ascribed as well to the protection and consolidation of international frontiers (Table 1), as asserting sovereignty and the possibilities of armed conflicts had a high relative weight in national politics in many new countries around the world (under autocratic governments or young democracies) (Zbicz & Green, 1997; Hegre, 2003). This motivation appeared to be especially influential in Latin America & Caribbean and South-east Asia & Oceania, where there is a large concentration of protected areas within the first hundreds of kilometers from borders (Fig. S2) and where there is a large fraction of post-independence protected areas (Table S2). How the change of these direct drivers might affect the relative strength of motivations and opportunistic forces remains to be explored, especially considering the transition of the promotion and deployment of protected areas from national governments to philanthropists and non-governmental organizations, or the empowerment of indigenous peoples or local rural populations (McNeely & Schutyser, 2003; Naughton-Treves, Holland & Brandon, 2005).

We should issue certain caveats from our analyses. First, the time dimension has not been explored, yet it could reveal important shifts in the strength of protection motivations and opportunistic forces (Joppa & Pfaff, 2009; Marinaro, Grau & Aráoz, 2012) and in the impact of evolving conservation paradigms (Mace, 2014). Second, the sampling approach implies a spatial integration of data into grid cells, and thus the results can mask heterogeneous biophysical or human conditions. For example, our analyses do not reveal the fact that some small protected areas that abut urban or productive areas were established under locally rough topographies and/or poor soils (e.g., Tijuca NP, Brazil; Sanjay Gandhi NP, India). An assessment focused on individual protected areas rather than on cells would solve this problem and would allow exploring the spatial dependencies in relation with the geometry of protected areas, as small and large areas may have different origins and geographical contexts (Andrew, Wulder & Coops, 2011). Third, our results are most probably affected by multicollinearity, as the explanatory variables of the distribution of protected areas are, by nature and in nature, correlated (e.g., cropland suitability derives—among other variables—from temperature). The applied random forest technique handles this phenomenon by means of the random selection of input variables at each node creation (*mtry*), but can not remove it completely (Breiman, 2001; Graham, 2003). Fourth, our study subjectively groups countries into regions and defines explanatory variables (biophysical, human, and biological) as proxies of individual motivations and opportunistic forces. Regarding the spatial grouping, even though the regions shared cultural, historical, and biogeographical traits, the proximate causes of protection (as defined above) and their consequences vary considerably within regions (e.g., Venezuela protecting 18.9% of its territory vs. Argentina protecting just 1.7%). Regarding the proxy variables, even though we considered the most up-to-date and accurate global information as far as we know, their selection could be modified, expanded, or improved with new or more suited options. For example, Durán et al. (2013) evaluated the representation within the Chilean protected network of different ecosystem services, including under this category the primary production, the carbon storage, the species richness, and the agricultural production. In this sense, our theoretical/methodological schemes can be subject to modifications and criticisms, and the precision and stability of our findings should be verified following alternative approaches.

## Conclusions

Present-day protected areas are mostly located in zones of relatively low productive value or population pressure, and to a lesser extent in areas of high tourism attractiveness. The search for geographical or biophysical representativeness and biodiversity conservation has had a relatively minor effect in shaping the distribution of land protection, in spite of their explicit priority in the debates and agendas of national and international conservation agencies. These geographical patterns will probably persist or increase (McNeely & Schutyser, 2003) under the concurrent expansion of protected networks (Jenkins & Joppa, 2009) and the increasing pressure on land resources (Foley et al., 2007; Ellis & Ramankutty, 2008). In this sense, representativeness and biodiversity conservation will only be strengthened if coupled with opportunistic forces. Operatively, this coupling requires a more explicit identification and spatial representation of conservation motivations (e.g., what are protection needs and targets of societies) and opportunities (e.g., where is it feasible to meet these needs and targets given current geographical and social conditions) (Andrew, Wulder & Coops, 2012; Martin et al., 2014). At last, if humans are increasingly considered as modelers and dependents of nature at regional and global levels (Van den Born et al., 2001; Lambin & Meyfroidt, 2011), future conservation policies will need to consider the role of goods and services like water provision, or tourism values (Durán et al., 2013) and the basic human need to interact with nature, which increases happiness and health, and fosters an environmentally sustainable behavior (Loreau, 2014; Zelenski & Nisbet, 2014).

##  Supplemental Information

10.7717/peerj.2989/supp-1 Supplemental Information 1 Supporting Information

## Additional Information and Declarations

### Data Availability

The following information was supplied regarding data availability: Baldi, German (2016): Baldi et al._Opportunities drive the global distribution of protected areas.xlsx. figshare.
https://dx.doi.org/10.6084/m9.figshare.4244777.v1.

## References

[ref-1] Akaike H (1974). A new look at the statistical model identification. IEEE Transactions on Automatic Control.

[ref-2] Allen RG, Pereira LS, Raes D, Smith MD (2004). Crop evapotranspiration. Guidelines for computing crop water requirements.

[ref-3] Andrew ME, Wulder MA, Coops NC (2011). Patterns of protection and threats along productivity gradients in Canada. Biological Conservation.

[ref-4] Andrew ME, Wulder MA, Coops NC (2012). Identification of de facto protected areas in boreal Canada. Biological Conservation.

[ref-5] Aycrigg JL, Davidson A, Svancara LK, Gergely KJ, McKerrow A, Scott JM (2013). Representation of ecological systems within the protected areas network of the continental United States. PLOS ONE.

[ref-6] Barr LM, Pressey RL, Fuller RA, Segan DB, McDonald-Madden E, Possingham HP (2011). A new way to measure the world’s protected area coverage. PLOS ONE.

[ref-7] Batjes N (2006). ISRIC-WISE derived soil properties on a 5 by 5 global grid (Version 1.1). Report 2006/02.

[ref-8] Belbin L (1993). Environmental representativeness: regional partitioning and reserve selection. Biological Conservation.

[ref-9] Bhagwat SA, Rutte C (2006). Sacred groves: potential for biodiversity management. Frontiers in Ecology and the Environment.

[ref-10] Bird K (2014). Ethnic quotas and ethnic representation worldwide. International Political Science Review.

[ref-11] Bourlière F, Adams AB (1962). Science and parks in the tropics. First world conference on national parks.

[ref-12] Breiman L (2001). Random forests. Machine Learning.

[ref-13] Brooks TM, Bakarr MI, Boucher T, Da Fonseca GAB, Hilton-Taylor C, Hoekstra JM, Moritz T, Olivieri S, Parrish J, Pressey RL, Rodrigues ASL, Sechrest W, Stattersfield A, Strahm W, Stuart SN (2004). Coverage provided by the global protected-area system: is it enough?. BioScience.

[ref-14] CIESIN-CIAT (2005). Gridded population of the world version 3 (GPWv3): population grids.

[ref-15] Costanza R, D’Arge  R, De Groot R, Farber S, Grasso  M, Hannon  B, Limburg K,  Naeem  S, O’Neill  RV, Paruelo  JM, Raskin  RG,  Sutton P, Van den Belt M (1997). The value of the world’s ecosystem services and natural capital. Nature.

[ref-16] Davis MB, Shaw RG (2001). Range shifts and adaptive responses to quaternary climate change. Science.

[ref-17] Durán AP, Casalegno S, Marquet PA, Gaston KJ (2013). Representation of ecosystem services by terrestrial protected areas: Chile as a case study. PLOS ONE.

[ref-18] Ellis EC, Kaplan JO, Fuller DQ, Vavrus S, Klein Goldewijk K, Verburg PH (2013). Used planet: a global history. Proceedings of the National Academy of Sciences of the United States of America.

[ref-19] Ellis EC, Ramankutty N (2008). Putting people in the map: anthropogenic biomes of the world. Frontiers in Ecology and the Environment.

[ref-20] Erize F (2003). El concepto de parque nacional en el mundo. Todo Es Historia.

[ref-21] FAO/IIASA (2011). Global agro-ecological zones (GAEZ V3.0).

[ref-22] Faraway JJ (2006). Extending the linear model with R: generalized linear, mixed effects and nonparametric regression models.

[ref-23] Foley JA, Monfreda C, Ramankutty N, Zaks D (2007). Our share of the planetary pie. Proceedings of the National Academy of Sciences of the United States of America.

[ref-24] Genuer R, Poggi JM, Tuleau-Malot C (2010). Variable selection using random forests. Pattern Recognition Letters.

[ref-25] Graham MH (2003). Confronting multicollinearity in ecological multiple regression. Ecology.

[ref-26] Grömping U (2009). Variable importance assessment in regression: linear regression versus random forest. The American Statistician.

[ref-27] Hegre H (2003). Disentangling democracy and development as determinants of armed conflict.

[ref-28] Hill MO (1973). Diversity and eveness: a unifying notation and its consequences. Ecology.

[ref-29] Hoekstra JM, Boucher TM, Ricketts TH, Roberts C (2005). Confronting a biome crisis: global disparities of habitat loss and protection. Ecology Letters.

[ref-30] Holdridge LR (1947). Determination of world plant formations from simple climatic data. Science.

[ref-31] Inglehart R, Welzel C (2005). Modernization, cultural change and democracy.

[ref-32] IUCN (1994). Guidelines for protected area management categories.

[ref-33] IUCN, UNEP-WCMC (2013). World database on protected areas (WDPA) annual release 2013 (web download version).

[ref-34] Jenkins CN, Joppa L (2009). Expansion of the global terrestrial protected area system. Biological Conservation.

[ref-35] Jenkins CN, Pimm SL, Joppa LN (2013). Global patterns of terrestrial vertebrate diversity and conservation. Proceedings of the National Academy of Sciences of the United States of America.

[ref-36] Joppa LN, Pfaff A (2009). High and far: biases in the location of protected areas. PLOS ONE.

[ref-37] Juffe-Bignoli D, Burgess ND, Bingham H, Belle EMS, De Lima MG, Deguignet M, Bertzky B, Milam AN, Martinez-Lopez J, Lewis E, Eassom A, Wicander S, Geldmann J, Van Soesbergen A, Arnell AP, O’Connor B, Park S, Shi YN, Danks FS, MacSharry B, Kingston N (2014). Protected planet report 2014.

[ref-38] Kareiva P, Marvier M (2003). Conserving biodiversity coldspots. American Scientist.

[ref-39] Kreft H, Jetz W (2007). Global patterns and determinants of vascular plant diversity. Proceedings of the National Academy of Sciences of the United States of America.

[ref-40] Lambin EF, Geist HJ, Lepers E (2003). Dynamics of land-use and land-cover change in Tropical Regions. Annual Review of Environment and Resources.

[ref-41] Lambin EF, Meyfroidt P (2011). Global land use change, economic globalization, and the looming land scarcity. Proceedings of the National Academy of Sciences of the United States of America.

[ref-42] Liaw A, Wiener M (2002). Classification and regression by randomforest. R News.

[ref-43] Loreau M (2014). Reconciling utilitarian and non-utilitarian approaches to biodiversity conservation. Ethics in Science and Environmental Politics.

[ref-44] Loucks C, Ricketts TH, Naidoo R, Lamoreux J, Hoekstra J (2008). Explaining the global pattern of protected area coverage: relative importance of vertebrate biodiversity, human activities and agricultural suitability. Journal of Biogeography.

[ref-45] Lovejoy TE (2006). Protected areas: a prism for a changing world. Trends in Ecology & Evolution.

[ref-46] Mace GM (2014). Whose conservation?. Science.

[ref-47] Maekawa M, Lanjouw A, Rutagarama E, Sharp D (2013). Mountain gorilla tourism generating wealth and peace in post-conflict Rwanda. Natural Resources Forum.

[ref-48] Margules CR, Pressey RL (2000). Systematic conservation planning. Nature.

[ref-49] Marinaro S, Grau HR, Aráoz E (2012). Extent and originality in the creation of national parks in relation to government and economical changes in Argentina. Ecología Austral.

[ref-50] Martin LJ, Quinn JE, Ellis EC, Shaw MR, Dorning MA, Hallett LM, Heller NE, Hobbs RJ, Kraft CE, Law E, Michel NL, Perring MP, Shirey PD, Wiederholt R (2014). Conservation opportunities across the world’s anthromes. Diversity and Distributions.

[ref-51] Mascia MB, Pailler S (2011). Protected area downgrading, downsizing, and degazettement (PADDD) and its conservation implications. Conservation Letters.

[ref-52] McDonald RI, Boucher TM (2011). Global development and the future of the protected area strategy. Biological Conservation.

[ref-53] McKercher B (1996). Differences between tourism and recreation in parks. Annals of Tourism Research.

[ref-54] McNeely JA, Harrison J, Dingwall PR (1994). Introduction: protected areas in the modern world. Protecting nature: regional reviews of protected areas.

[ref-55] McNeely JA, Schutyser F (2003). Protected Areas in 2023: Scenarios for an Uncertain Future. Vth World Congress on Protected Areas.

[ref-56] Mulholland G, Eagles PFJ (2002). African parks: combining fiscal and ecological sustainability. Parks.

[ref-57] Myers N, Mittermeier RA, Mittermeier CG, Da Fonseca GAB, Kent J (2000). Biodiversity hotspots for conservation priorities. Nature.

[ref-58] Naughton-Treves L, Holland MB, Brandon K (2005). The role of protected areas in conserving biodiversity and sustaining local livelihoods. Annual Review of Environment and Resources.

[ref-59] Nelson A (2008). Estimated travel time to the nearest city of 50,000 or more people in year 2000.

[ref-60] New M, Lister D, Hulme M, Makin I (2002). A high-resolution data set of surface climate over global land areas. Climate Research.

[ref-61] Olson DM, Dinerstein E (2002). The Global 200: priority ecoregions for global conservation. Annals of the Missouri Botanical Garden.

[ref-62] Paül Carril V, Santos Solla XM, Pazos Otón M (2015). The ambiguous geographies of protected areas in Galicia. Ambiente Y Desarrollo.

[ref-63] Pressey RL (1994). Ad hoc reservations: forward or backward steps in developing representative reserve systems?. Conservation Biology.

[ref-64] Pyle RM (2003). Nature matrix: reconnecting people and nature. Oryx.

[ref-65] Rodrigues ASL, Akçakaya HR, Andelman SJ, Bakarr MI, Boitani L, Brooks TM, Chanson JS, Fishpool LDC, Da Fonseca GAB, Gaston KJ, Hoffmann M, Marquet PA, Pilgrim JD, Pressey RL, Schipper J, Sechrest W, Stuart SN, Underhill LG, Waller RW, Watts MEJ, Yan X (2004a). Global gap analysis: priority regions for expanding the global protected-area network. BioScience.

[ref-66] Rodrigues ASL, Andelman SJ, Bakan MI, Boitani L, Brooks TM, Cowling RM, Fishpool LDC, Da Fonseca GAB, Gaston KJ, Hoffmann M, Long JS, Marquet PA, Pilgrim JD, Pressey RL, Schipper J, Sechrest W, Stuart SH, Underhill LG, Waller RW, Watts MEJ, Yan X (2004b). Effectiveness of the global protected area network in representing species diversity. Nature.

[ref-67] Ruesch A, Gibbs HK (2008). New IPCC Tier-1 global biomass carbon map for the year 2000.

[ref-68] SCBD (2010). COP-10 Decision X/2.

[ref-69] Scott JM (1993). Gap Analysis: a geographic approach to protection of biological diversity. Wildlife Monographs.

[ref-70] Scott D, Malcolm JR, Lemieux C (2002). Climate change and modelled biome representations in Canada’s national park system. Global Ecology & Biogeography.

[ref-71] Sellars RW (1997). Preserving nature in the national parks: a history.

[ref-72] Siikamäki P, Kangas K, Paasivaara A, Schroderus S (2015). Biodiversity attracts visitors to national parks. Biodiversity and Conservation.

[ref-73] Sodhi NS, Koh LP, Brook BW, Ng PKL (2004). Southeast Asian biodiversity: an impending disaster. Trends in Ecology & Evolution.

[ref-74] Stattersfield AJ, Crosby MJ, Long AJ, Wege DC (1998). Endemic bird areas of the world. Priorities for biodiversity conservation.

[ref-75] Szafer W, Michajlow  W (1973). History of nature conservation in the world and in Poland. Protection of man’S natural environment: a collective work.

[ref-76] Terborgh J, Winter B (1983). A method for siting parks and reserves with special reference to Columbia and Ecuador. Biological Conservation.

[ref-77] UK National Parks (2015). National Park facts and figures. http://www.nationalparks.gov.uk/.

[ref-78] USGS (2004). SRTM elevation data. University of Maryland.

[ref-79] Van den Born RJG, Lenders RHJ, De Groot WT, Huijsman E (2001). The new biophilia: an exploration of visions of nature in Western countries. Environmental Conservation.

[ref-80] Venter O, Fuller RA, Segan DB, Carwardine J, Brooks T, Butchart SHM, Di Marco M, Iwamura T, Joseph L, O’Grady D, Possingham HP, Rondinini C, Smith RJ, Venter M, Watson JEM (2014). Targeting global protected area expansion for imperiled biodiversity. PLOS Biology.

[ref-81] Vitousek PM, Mooney HA, Lubchenco J, Melillo JM (1997). Human domination of Earth’s ecosystems. Science.

[ref-82] Watson JEM, Dudley N, Segan DB, Hockings M (2014). The performance and potential of protected areas. Nature.

[ref-83] Whittaker RJ (1987). An application of Detrended Correspondence Analysis and Non-Metric Multidimensional scaling to the identification and analysis of environmental factor complexes and vegetation structures. Journal of Ecology.

[ref-84] Wirth CL, Adams AB (1962). National parks. First world conference on national parks.

[ref-85] Zbicz DC, Green MJB (1997). Status of the world’s transfrontier protected areas. Parks.

[ref-86] Zelenski JM, Nisbet EK (2014). Happiness and feeling connected: the distinct role of nature relatedness. Environment and Behavior.

