# Supplementary material for: Opportunities drive the global distribution of protected areas
Source: PeerJ. 2017 Feb 15;5:e2989. doi: 10.7717/peerj.2989 (PMC5314958; doi:10.7717/peerj.2989)
Supplement: Supplemental Information 1 [file peerj-05-2989-s001.doc]

# Supporting Information

Table S1.

Title: Samples by region and globe.

Legend: Number of 0.5° square cells samples, by region and globe.

| **Region** | **Samples** |
| --- | --- |
| Latin America & Caribbean | 7,607 |
| North America & Australia–NZ | 18,602 |
| Sub-Saharan Africa | 8,430 |
| Middle East & North Africa | 4,725 |
| West Europe | 3,462 |
| East Europe & Central Asia | 15,337 |
| South-east Asia & Oceania | 8,392 |
| Global | 66,555 |


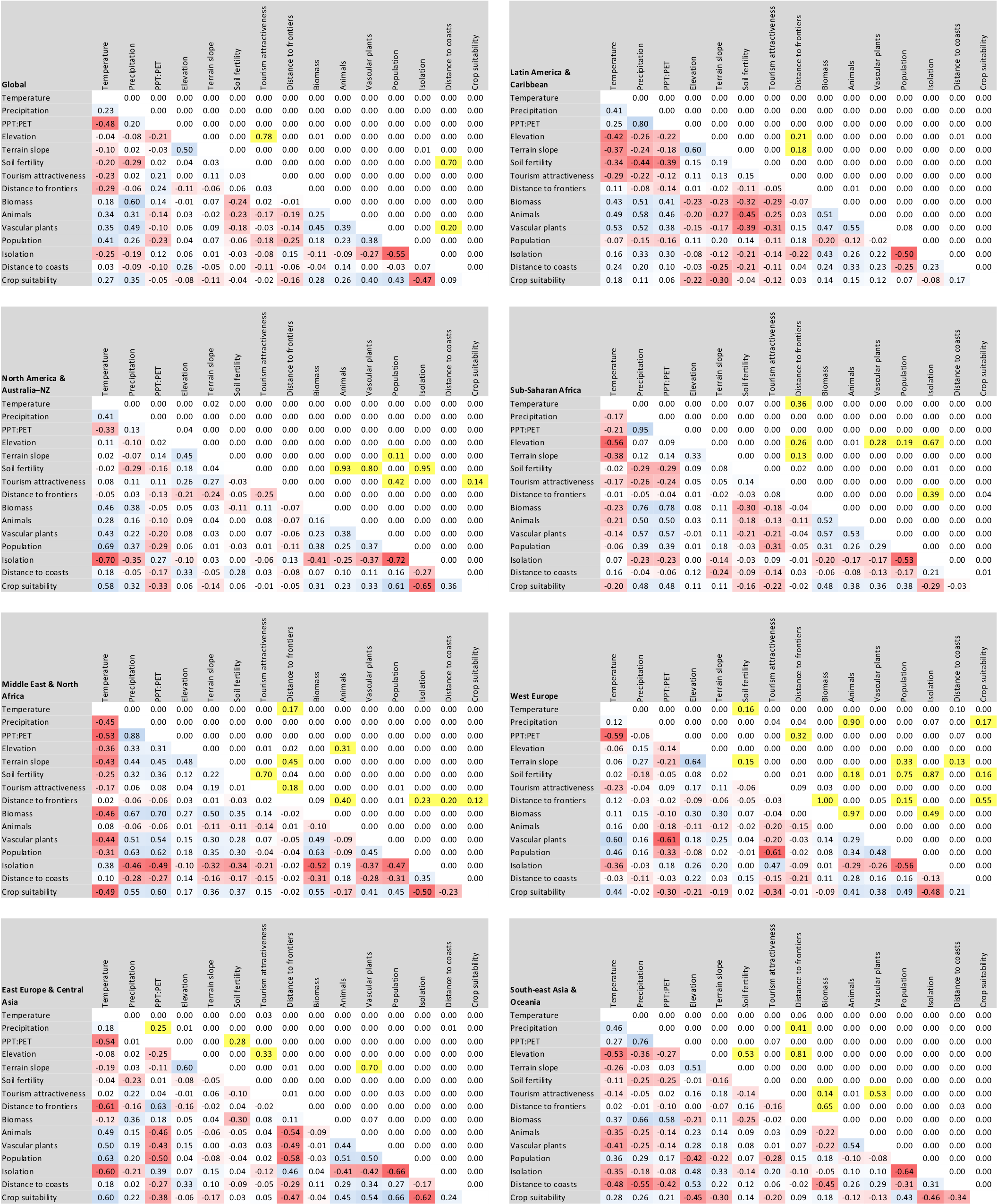


Figure S1.

Title: Correlations between the 15 independent variables.

Legend: Kendall’s correlation coefficients (τ, lower diagonal) and their significance level (*p*-*value*, upper diagonal) at global and regional levels; colors represents strength and sign of the correlation (from negative red, to white, to positive blue), or a *p*-*value* > 0.1 (in yellow).





Figure S2.

Title: Regional distribution of protected areas.

Legend: Regional distribution of protected areas along biophysical, human, and biological gradients. Histograms depict the regional area in each class of the 15 independent variables (light gray bars), the area under protection in each class (intervals in the histograms) of the independent variable (dark gray bars), and the fraction under protection of the class of the independent variable (red dots and lines). Lower and upper classes were grouped using the percentile values 0.025 and 0.975 of the independent variable. Blue asterisks denote that histograms are generated with the log_10_ transformed independent variable, and thus do not correspond with the same data used for the statistical analyses. Green asterisks denote that classes in histograms were divided in order reach ≥8 *j* intervals in the statistical analyses. In order or appearance (and shown by inset maps): Latin America & Caribbean, North America & Australia–NZ, Sub-Saharan Africa, Middle East & North Africa, West Europe, East Europe & Central Asia, and South-east Asia & Oceania.





Figure S2. cont.





Figure S2. cont.





Figure S2. cont.





Figure S2. cont.





Figure S2. cont.





Figure S2. cont.


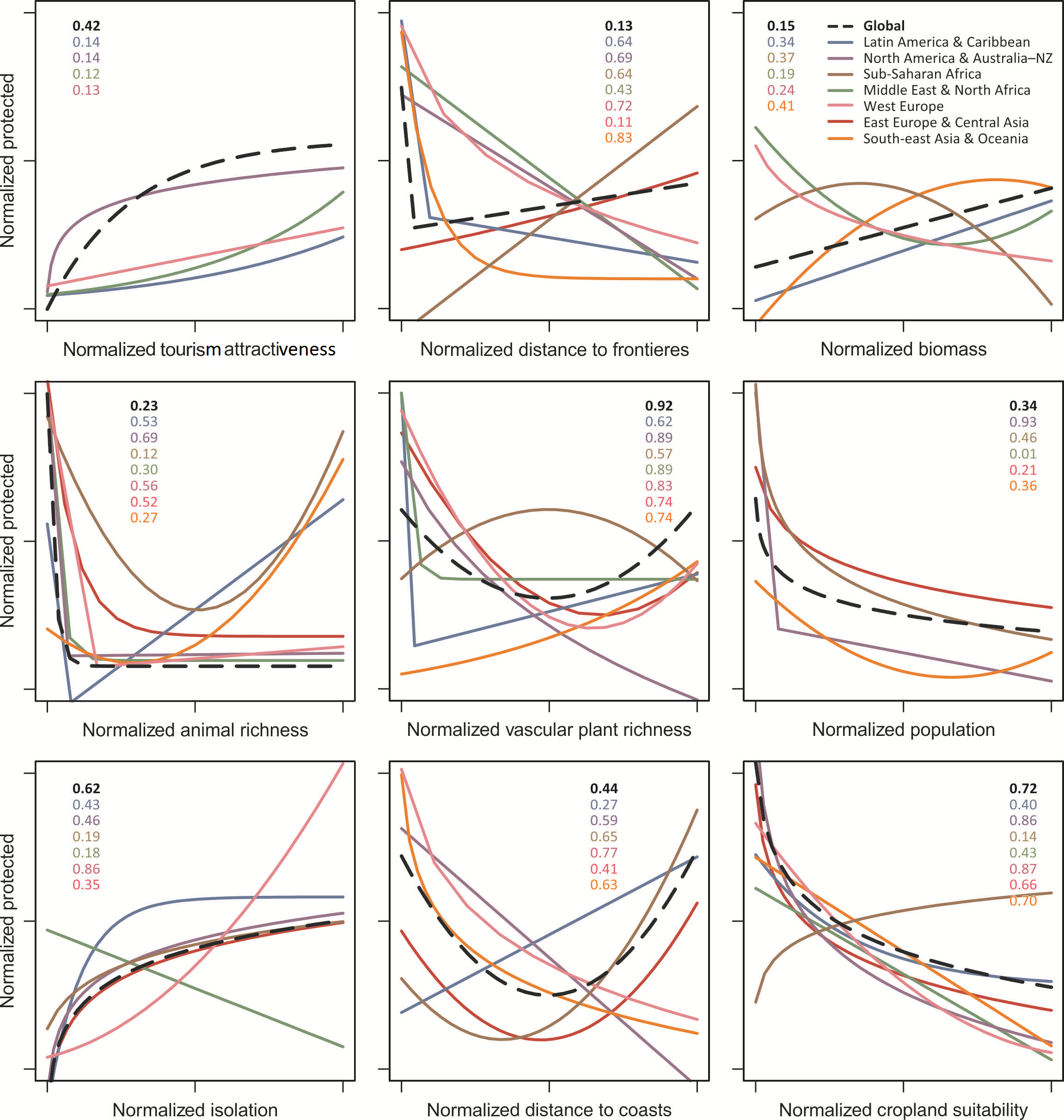


Figure S3.

Title: Regression models of the fraction under protection along human and biological gradients.

Legend: Regression models of the fraction under protection along human and biological variables (red lines in Fig. S2) related to preferential motivations and opportunistic forces, at global and regional levels. Numbers represent pseudo-R^2^ values. Models with a pseudo-R^2^ < 0.1 were not plotted for simplification purposes. Plot axes were normalized using the ranges of the values of fraction and of the independent variables.

Table S2.

Title: Regional cultural and conservation aspects.

Legend: General cultural and conservation aspects across regions. Variables 1-5 came from averaging national level statistical data, weighted by country area. Sources: gross domestic product (GDP) and income per person (The World Bank 2014); Human Development Index (UNPD 2014); survival vs. self-expression values and traditional vs. secular-rational values (WVS 2014); national independence day (www.wikipedia.org); protected area (PA) downgrading, downsizing, and degazettement (PADDD) (World Wildlife Fundation 2014); protected areas budget (James et al. 1999); data concerning protected areas (IUCN and UNEP-WCMC 2013). Variables 4 and 5 are unitless, and when higher the values, the higher the relative importance of self-expression or secular-rational values.

|  | **Latin America & Caribbean** | **North America & Australia–NZ** | **Sub-Saharan Africa** | **Middle East & North Africa** | **West Europe** | **East Europe & Central Asia** | **South-east Asia & Oceania** |
| --- | --- | --- | --- | --- | --- | --- | --- |
| GDP (2014 US$, 2006-2012) | 9.90 x 10^11^ | 6.06 x 10^12^ | 4.72 x 10^10^ | 2.52 x 10^11^ | 9.25 x 10^11^ | 1.11 x 10^12^ | 3.12 x 10^12^ |
| Income per person (2000 US$, 2006-2011) | 5030 | 29641 | 780 | 4838 | 19959 | 2515 | 2490 |
| Human Development Index (2013) | 0.75 | 0.92 | 0.47 | 0.72 | 0.87 | 0.76 | 0.67 |
| Survival vs. self-expression values (2010-2014) | 0.41 | 1.70 | -0.18 | -0.76 | 0.65 | -1.18 | -0.58 |
| Traditional vs. secular-rational values (2010-2014) | -0.96 | -0.02 | -0.93 | -0.99 | 0.63 | 0.43 | 0.60 |
| Date of first protected area | 1907 | 1872 | 1895 | 1933 | 1838 | 1819 | 1889 |
| PA pre-formation of the modern state (%) | 2.7 | 35.3 | 44.3 | 0.9 | 31.8 | 54.3 | 2.4 |
| Protected area (%) | 6.6 | 11.4 | 6.3 | 2.1 | 5.1 | 6.8 | 3.2 |
| Protected area (number) | 1646 | 24538 | 771 | 219 | 41805 | 3447 | 1467 |
| PA budget relative to GDP (‰, 1990-2000) | 0.11 | 0.32 | 0.49 | 0.05 | 0.18 | nd | 0.11 |
| PADDD vs. total protected (‰) | 2.5 | 26.1 | 9.3 | 0.0 | 0.0 | 6.0 | 1.0 |

Table S3.

Title: Protected fraction (%) along each biophysical gradient.

Legend: Average of the fraction under protection (%) for the intervals (in the histograms) of each biophysical independent variable at global and regional levels. Italics indicate a fraction under protection ≥ 17, indicating an achievement of the Aichi Biodiversity target 11 (Strategic Plan 2011-2020).

|  | **Global** | **Latin America & Caribbean** | **North America & Australia–NZ** | **Sub-Saharan Africa** | **Middle East & North Africa** | **West Europe** | **East Europe & Central Asia** | **South-east Asia & Oceania** |
| --- | --- | --- | --- | --- | --- | --- | --- | --- |
| Temperature | 8.5 | 5.3 | 14.0 | 6.4 | 1.9 | *17.6* | 6.6 | 2.4 |
| Precipitation | 7.2 | 7.7 | 13.2 | 6.0 | 1.3 | 10.1 | 7.2 | 4.7 |
| PPT:PET | 7.2 | 7.5 | 11.9 | 6.1 | 1.3 | 4.3 | 5.5 | 4.0 |
| Elevation | 9.2 | 6.5 | *17.6* | 5.9 | 2.6 | 7.7 | 7.9 | 3.1 |
| Terrain slope | 8.3 | 7.9 | *17.4* | 6.3 | 2.5 | 7.7 | 9.4 | 4.0 |
| Soil fertility | 5.7 | 4.9 | 8.5 | 6.0 | 2.1 | 4.9 | 6.1 | 3.1 |

References

IUCN and UNEP-WCMC. 2013. World Database on Protected Areas (WDPA) Annual Release 2013 (web download version). Cambridge, UK.

James AN, Green MJ, Beverley, and Paine JR. 1999. A Global Review of Protected Area Budgets and Staff. WCMC Biodiversity Series No10. Cambridge, UK: IUCN World Commission on Protected Areas (WCPA) - WCMC. p 46.

The World Bank. 2014. World Development Indicators.

UNPD. 2014. Human Development Report 2014. Sustaining Human Progress: Reducing Vulnerabilities and Building Resilience. New York, USA: United Nations Development Programme. p 226.

World Wildlife Fundation. 2014. PADDDtracker.org Data Release Version 1.0. World Wildlife Fundation.

WVS. 2014. World Values Survey Association (www.worldvaluessurvey.org). Wave 6 2010-2014 Aggregate File Producer: Asep/JDS. OFFICIAL AGGREGATE v.20150418. Madrid, Spain.
